# Cytotoxicity of Triterpene *Seco*-Acids from *Betula pubescens* Buds

**DOI:** 10.3390/molecules24224060

**Published:** 2019-11-09

**Authors:** Łukasz Szoka, Valery Isidorov, Jolanta Nazaruk, Marcin Stocki, Leszek Siergiejczyk

**Affiliations:** 1 Department of Medicinal Chemistry, Medical University of Bialystok, 15-222 Białystok, Poland; lukasz.szoka@umb.edu.pl; 2 Forest Faculty, Białystok University of Technology, 17-200 Hajnówka, Poland; m.stocki@pb.edu.pl; 3 Department of Pharmacognosy, Medical University of Bialystok, 15-222 Białystok, Poland; jolanta.nazaruk@umb.edu.pl; 4 Institute of Chemistry, University of Białystok, 15-245 Białystok, Poland; nmrbial@uwb.edu.pl

**Keywords:** birch buds, triterpene *seco*-acids, cytotoxicity, apoptosis

## Abstract

The present study investigated the magnitude and mechanism of the cytotoxic effect on selected cancer cell lines of 3,4-*seco*-urs-4(23),20(30)-dien-3-oic acid (**1**), 3,4-*seco*-olean-4(24)-en-19-oxo-3-oic acid (**2**), and 3,4-*seco*-urs-4(23),20(30)-dien-19-ol-3-oic acid (**3**) isolated from downy birch (*Betula pubescens*) buds by carbon dioxide supercritical fluid extraction and gradient column chromatography. Cell viability in six human cancer lines exposed to these compounds was determined by 3-(4,5-dimethylthiazol-2-yl)-2,5-diphenyltetrazolium bromide (MTT) assay. Apoptosis was quantified by annexin V/propidium iodide staining of gastric cancer AGS and colorectal cancer DLD-1 cells. To evaluate the mechanism of apoptosis, the expression of apoptosis-related proteins was analyzed by Western blot. Compound **1** exhibited non-specific toxicity, while compounds **2** and **3** were specifically toxic to colon and stomach cancer cells. The toxicity of compounds **2** and **3** against these two cell lines was greater than for compound **1**. Cleavage of caspase-8, -9, and -3 was found in AGS and DLD-1 cells treated with all three *seco*-acids, indicating the induction of apoptosis via extrinsic and intrinsic pathways. Therefore, triterpene *seco*-acids (**1**–**3**) decreased cell viability by apoptosis induction. AGS and DLD-1 cells were more susceptible to *seco*-acids with an oxidized C19 than normal fibroblasts. Hence, it made them a new group of triterpenes with potential anticancer activity.

## 1. Introduction

Birch trees are deciduous hardwood species from the *Betulaceae* family and are widespread in the Northern Hemisphere. Since ancient times, different parts of these plants have been used in traditional medicine [1,2]. In particular, ethanol tinctures of winter buds have been used to treat toothache, colds, and rheumatoid conditions. A number of laboratory and clinical studies have confirmed the therapeutic properties of birch bud extracts, including their diuretic action as well as antiseptic and antioxidant properties [3,4,5,6]. In Russia, a birch bud preparation, Gemmae Betulae, is a standardized medicine used mainly to treat urinary tract diseases [7].

In recent decades, a number of studies have been conducted on the anti-cancer activity of birch buds [8,9,10,11]. In particular, it was found that various extracts from the buds of two species of white birch exhibited distinct time- and concentration-dependent cytotoxicity with respect to many human cancer cell lines [10,11]. At the same time, the highest cytotoxic activity was demonstrated by extracts obtained by carbon dioxide supercritical fluid extraction (SFE). These results suggest that birch buds are a promising source of compounds with cytotoxic activity against various forms of cancer. Notably, birch buds have a high content of triterpene compounds [12,13]. Many members of this group of secondary metabolites have well-documented anti-cancer activity [14,15,16]. However, to date, research has been limited to the action of whole extracts obtained from birch buds, and no attempt has been made to study the anti-cancer effect of individual constituent compounds.

**Figure 1:**
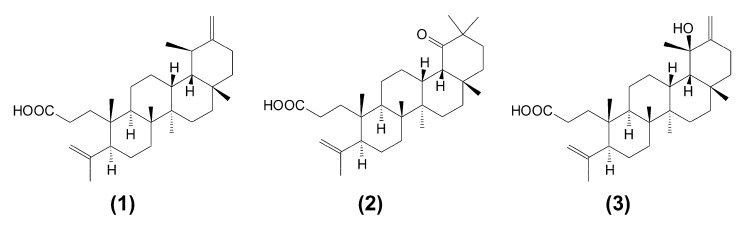
Chemical structure of triterpene *seco*-acids: 3,4-*seco*-urs-4(23),20(30)-dien-3-oic acid (**1**), 3,4-*seco*-olean-4(24)-en-19-oxo-3-oic acid (**2**), and 3,4-*seco*-urs-4(23),20(30)-dien-19-ol-3-oic acid (**3**).

This work reports on the cytotoxic effect of triterpene *seco*-acids (**1**–**3**), which were isolated for the first time from buds of downy birch, *Betula pubescens*, as seen in Figure 1.

## 2. Results

### 2.1. Triterpene Seco-Acids Decreased Viability of Cancer Cells

**Figure 2:**
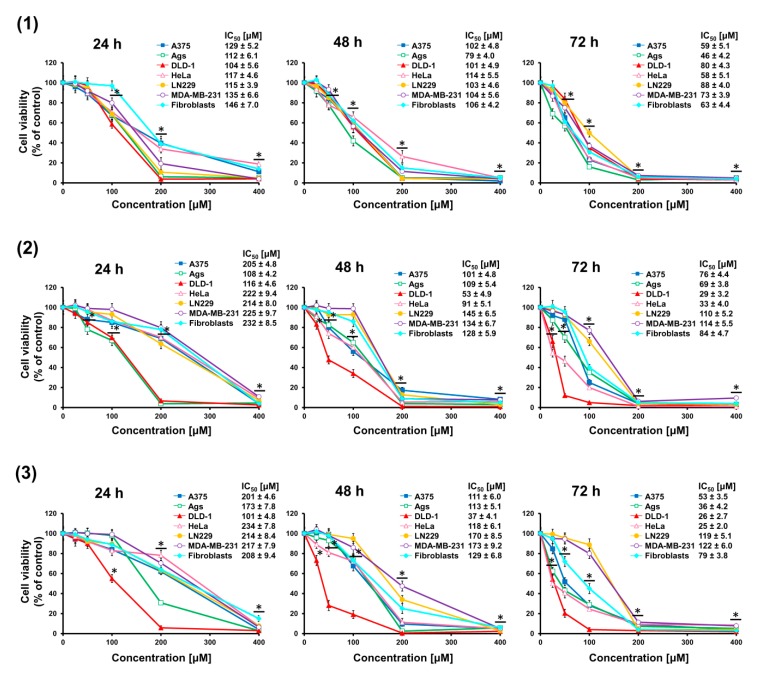
Triterpene *seco*-acids decrease cell viability. Effect of 3,4-*seco*-urs-4(23),20(30)-dien-3-oic acid (**1**), 3,4-*seco*-olean-4(24)-en-19-oxo-3-oic acid (**2**), and 3,4-*seco*-urs-4(23),20(30)-dien-19-ol-3-oic acid (**3**) on the viability of cancer cells A375, AGS, DLD-1, HeLa, LN229, MD-MB-231 and normal dermal fibroblasts after 24, 48, and 72 h treatment, as assessed by MTT assay. Data are presented as mean ± standard error of the mean (SEM) from three independent experiments. * *p* < 0.05 compared to control group. The half-maximal inhibitory concentration (IC_50_) values of *seco*-acids for the cell lines are shown.

Cancer cells A375, AGS, DLD-1, HeLa, LN229, MDA-MB-231, and normal skin fibroblasts were treated with increasing doses (25–400 μM) of *seco*-acids (compounds **1**–**3**) for 24, 48, and 72 h, and changes in cell viability were quantified using the MTT assay, as depicted in Figure 2. A dose- and time-dependent decrease in cell viability was observed. Based on the dose–response curves, the IC_50_ values were calculated. It was found that the decrease in viability of cells treated with *seco*-acid (**1**) was similar. By contrast, bifunctional compounds keto-*seco*-acid (**2**) and hydroxy-*seco*-acid (**3**) exerted higher activity against DLD-1 cells. In addition, DLD-1 cells were more sensitive to the action of these *seco*-acids than normal fibroblasts. The most relevant IC_50_ values are presented in Table 1.

### 2.2. Triterpene Seco-Acids Increased Apoptosis in AGS and DLD-1 Cells

**Figure 3:**
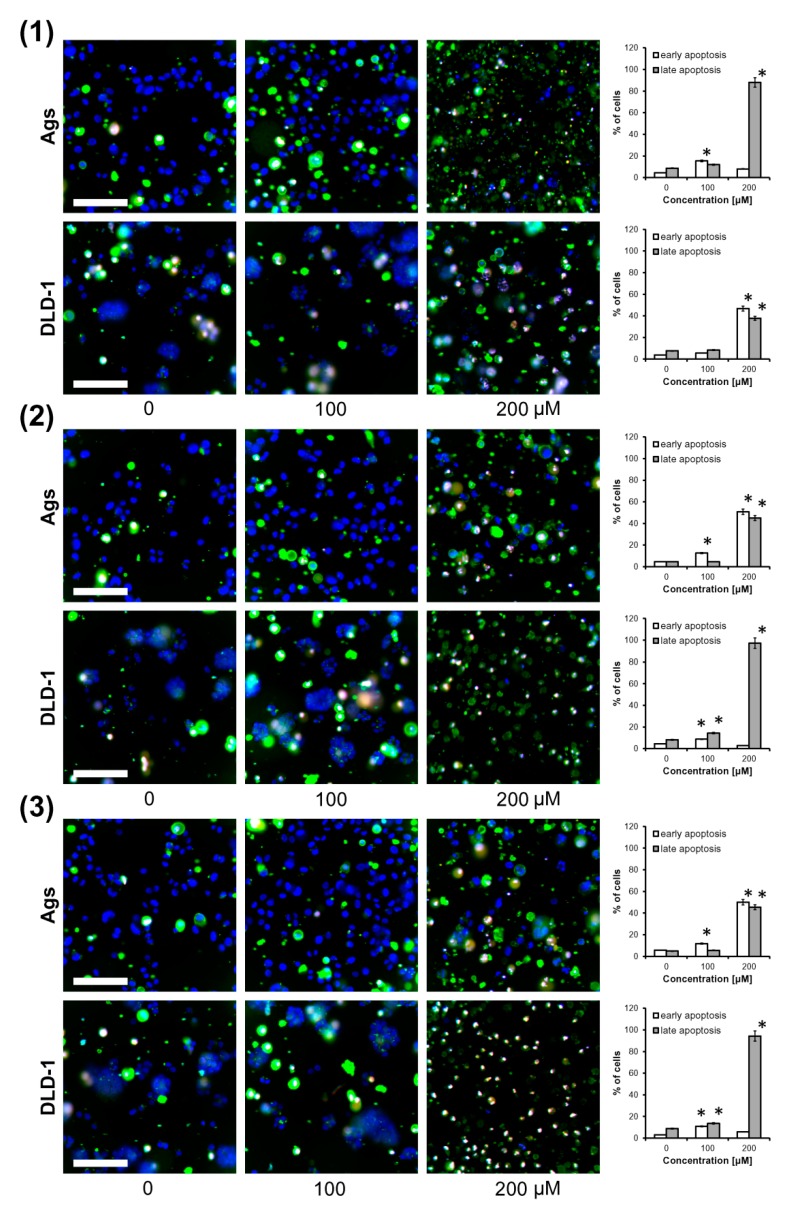
Triterpene *seco*-acids promote apoptosis in AGS and DLD-1 cells. AGS and DLD-1 cells treated with 3,4-*seco*-urs-4(23),20(30)-dien-3-oic acid (**1**), 3,4-*seco*-olean-4(24)-en-19-oxo-3-oic acid (**2**), and 3,4-*seco*-urs-4(23),20(30)-dien-19-ol-3-oic acid (**3**) for 24 h were triple stained with annexin V-FITC conjugate (green fluorescence), propidium iodide (red fluorescence), and Hoechst 33342 (blue fluorescence), and were visualized by fluorescence microscopy. Representative merged photographs are shown. Scale bar = 100 μm. Data are presented as mean ± standard deviation (SD) from three assays. * *p* < 0.05 compared to control group.

Since birch bud extracts were used in Russian medicine as a diuretic agent taken by mouth [2,3], gastric cancer cells (AGS) and colorectal cancer cells (DLD-1) were chosen for the examination of the apoptotic activity of triterpene *seco*-acids isolated from them. Apoptosis was quantified by triple staining of cells with annexin V-FITC conjugate, propidium iodide (PI), and Hoechst 33342, and cells were visualized by fluorescent microscopy, as depicted in Figure 3. Apoptotic cells were characterized by the exposure of phosphatidylserine on the outer leaflet of the cellular membrane. Thus, annexin V, a natural ligand of phosphatidylserine, was used for their detection. Membrane integrity in early apoptotic cells made them impermeable to PI; however, DNA of late apoptotic or dead cells could bind it. In contrast to PI, Hoechst 33342 had the ability to bind the DNA of living and dead cells. We found that after 24 h of treatment, triterpene *seco*-acids increased apoptotic cell death in a dose-dependent manner. Compound **1** at 200 μM induced a late apoptotic stage in almost all AGS cells, while the number of early and late apoptotic DLD-1 cells was similar. In contrast, treatment of AGS cells with compounds **2** and **3** at 200 μM resulted in a similar increase in the number of early and late apoptotic cells, and put almost all DLD-1 cells into a late apoptotic stage.

### 2.3. Triterpene Seco-Acids Activated Extrinsic and Intrinsic Pathway of Apoptosis in AGS and DLD-1 Cells

**Figure 4:**
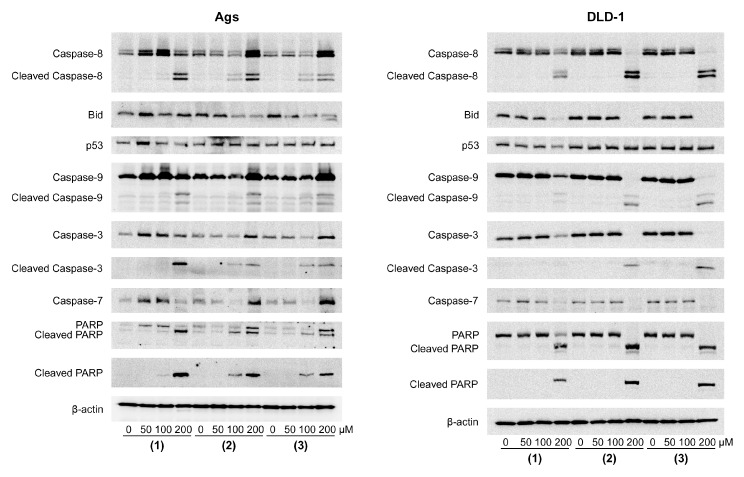
Triterpene *seco*-acids trigger extrinsic and intrinsic pathways of apoptosis in AGS and DLD-1 cells. AGS and DLD-1 cells were treated with 3,4-*seco*-urs-4(23),20(30)-dien-3-oic acid (**1**), 3,4-*seco*-olean-4(24)-en-19-oxo-3-oic acid (**2**), and 3,4-*seco*-urs-4(23),20(30)-dien-19-ol-3-oic acid (**3**) for 24 h, and the expression levels of apoptosis-related proteins were determined by Western blotting. Antibodies against caspase-8, BID, p53, caspase-9, caspase-3, cleaved caspase-3, caspase-7, PARP and cleaved PARP were used. The expression of β-actin served to normalize protein loading.

To evaluate the mechanism of apoptosis induction by triterpene *seco*-acids in AGS and DLD-1 cells, the expression of apoptosis-related proteins was analyzed by Western blot, as shown in Figure 4. The final stage of apoptosis was carried out by caspases—a group of cysteine-aspartyl-specific proteases. Activation of caspases depended on intrachain cleavage of inactive zymogens to form active cleaved caspases. There were two major apoptosis-triggering pathways: extrinsic and intrinsic.

The extrinsic pathway was activated by ligands of transmembrane death receptors, resulting in activation of caspase-8. Increase in caspase-8 processing was observed by all triterpene *seco*-acids in both cell lines used in the study, with the data suggesting induction of the extrinsic apoptosis pathway.

Multiple internal stimuli are known to trigger the intrinsic pathway of apoptosis by increasing the permeability of outer mitochondrial membranes. This resulted in translocation of cytochrome c into the cytoplasm and caspase-9 activation. Among intrinsic pathway-inducing proteins, expression levels of BID and p53 were assayed. BID was previously shown to be cleaved by caspase-8 to form truncated BID (tBID), which translocated into mitochondrial membranes, resulting in cytochrome c release. Our data showed a decrease in BID expression in both lines, except for *seco*-acid (**1**)-treated AGS cells. By contrast, there was no increase of p53 level in wild-type p53-containing AGS cells or in DLD-1 cells carrying an inactivating mutation in the TP53 gene [17]. Thus, BID but not p53 could mediate induction of the intrinsic pathway. Finally, we found an increase in caspase-9 cleavage in both cell lines treated with all three triterpene *seco*-acids, indicating intrinsic pathway induction.

Both apoptosis pathways were previously shown to converge in the induction of executioner caspases, such as caspase-3 and caspase-7, in turn catalyzing the proteolytic degradation of many cellular components, leading to cell death. Treatment with the three triterpene *seco*-acids induced an increase in active cleaved caspase-3 levels in AGS and DLD-1 cells with a concomitant decrease in expression of caspase-3 pro-form in DLD-1 cells. Although cleaved caspase-7 was not detected in DLD-1 cells after 24 h of treatment, the observed decrease in its pro-form level indicates caspase-7 activation. In contrast, treatment of AGS cells with *seco*-acids resulted in not only increased levels of cleaved caspase-3 but also increased expression of executioner caspase pro-forms. Cleavage of poly(ADP-ribose) polymerase (PARP), the substrate for caspase-3, was concomitant with caspase-3 processing in both cell lines, indicating enzymatic activation of caspase-3. Taken together, these data suggest the activation of executioner caspases through the extrinsic and intrinsic pathways of apoptosis. The highest levels of cleaved caspase-8, -9, and -3 and cleaved PARP were observed in AGS cells treated with compound **1**, and in DLD-1 cells treated with compounds **2** and **3**. Thus, cells recognized as late apoptotic on annexin V and PI staining had higher levels of apoptosis markers.

## 3. Discussion

The genus *Betula* is a rich source of triterpenes that exert cytotoxic activity and are thus considered as potential anticancer agents, with betulinic acid being a well-known example [18]. In turn, less-active compounds such as betulin, which is the major constituent of the outer bark of birch trees, are used for obtaining semi-synthetic derivatives with promising activity [19,20]. Triterpene biosynthesis pathways vary depending on the plant organ [21]; hence, searching for triterpenes is not limited to birch bark. Indeed, the presence of both ursane and oleanane triterpenes was reported in floral spikes of *Betula platyphylla* [16]. Triterpenes belonging to the oleanane and lupane groups, besides ursane derivatives, are also of interest due to their possible use in cancer therapy [15].

Birch buds seem to be another interesting source of triterpenes. In a recent paper [11], the cytotoxicity of various birch bud extracts against a wide range of cancer cells was demonstrated. In the current study, we isolated triterpene *seco*-acids (compounds **1**–**3**) from *B. pubescens* buds and determined their cytotoxicity to cancer cells and normal fibroblasts.

One of the isolated compounds, *seco*-acid **1** was previously reported to show weak cellular toxicity in multiple cell lines [16]. Our data supported these findings; however, we also demonstrated a time dependency in all tested cell lines. Moreover, the addition of a hydroxyl group to C19 of *seco*-acid **1** caused an increase in cytotoxicity in some of the cell lines—particularly in colorectal and gastric cancer cells. Therefore, the incorporation of polar groups into ursane type *seco*-acids, as in compound **3**, might have a beneficial impact on the possible application range. Additionally, it seemed that this feature was not unique to ursane-type *seco*-acids, because oleanane type derivative **2**, characterized by a keto group at C19, retained high cytotoxicity in colorectal cancer culture. This was consistent with an earlier report that an increase in the polarity of triterpenes causes an increase in anticancer activity [22].

Cancer cells are characterized by apoptosis resistance, and apoptosis induction is a common mechanism of anticancer drugs [23]. Apoptosis is a highly regulated process characterized by membrane blebbing, chromatin condensation, and cell shrinkage [24]. One of the most important events defining cell fate is the loss of phospholipid asymmetry with the externalization of phosphatidylserine, which functions as a chemical signal for phagocytosis [24]. Oleanane and ursane triterpenes have been shown to promote apoptosis in many cancer cells [15,16]. In the current study, both colorectal and gastric cancer cells treated with triterpene *seco*-acids showed externalization of phosphatidylserine, indicating apoptosis. Moreover, to different extents, cells lost membrane integrity—a phenomenon common to the late stage of apoptosis or necrosis.

The majority of apoptosis events are driven by caspases. These members of the cysteine proteases exist in the cytoplasm as zymogens [24]. Executioner caspases (e.g., caspase-3 and -7) catalyze the degradation of enzymes and structural proteins. The activation of executioner caspases is regulated by initiator caspases (e.g., caspase-8 and -9). Oleanane and ursane triterpenoids have been reported to activate the intrinsic pathway of apoptosis [15]. In addition, the three triterpene *seco*-acids examined in the current study acted as activators of caspase-9, which was a mediator of the intrinsic pathway [25]. Consequently, activation of caspase-3, -7 and degradation of PARP occurred in DLD-1 and AGS cells. Notably, the level of p53 was unchanged in both cell lines; however, BID was down-regulated. tBID serves as a switch for activation of the intrinsic pathway by caspase-8, which in turn acts as the main intermediate of the extrinsic pathway [26,27]. It was reported that triterpenes can promote apoptosis via the extrinsic pathway [28]. Indeed, activation of caspase-8 by all three triterpene *seco*-acids was observed in the current study. Accordingly, apoptosis induced by the triterpene *seco*-acids was the result of the activation of both extrinsic and intrinsic pathways.

In conclusion, this study showed that triterpene *seco*-acids decreased cell viability by apoptosis induction in gastric and colorectal cancer cells. Furthermore, these cancer cells were more susceptible to the toxicity of *seco*-acids when they had an oxidized C19, compared to normal fibroblasts. As such, they might be considered for further investigations as a novel group of drug candidates for the treatment of colorectal cancer.

## 4. Materials and Methods 

### 4.1. Reagents and Chemicals

Stationary phases and eluents used in column chromatography and extraction were: silica gel (mesh 0.063–0.2 mm, Merck, Darmstadt, Germany); polyamide (Roth, Karlsrue, Germany); *n*-hexane, diethyl ether, benzene, and chloroform (Chempur, Pekary Śląskie, Poland); methanol, ethyl acetate, and acetone (POCH, Gliwice, Poland).

Antibody against β-actin (A2066, dilution 1:2000), horseradish peroxidase-conjugated secondary antibodies, anti-rabbit (A9169, dilution 1:5000) and anti-mouse (A9044, dilution 1:5000), and 3-(4,5-dimethylthiazole-2-yl)-2,5-diphenyltetrazolium bromide (MTT) were obtained from Sigma-Aldrich (Poznań, Poland). Dulbecco’s modified Eagle’s medium (DMEM), penicillin, streptomycin, fetal bovine serum (FBS), phosphate-buffered saline, trypsin, and Hoechst 33342 were purchased from Thermo Fisher Scientific (Warszawa, Poland). Antibodies against caspase-3 (9662, dilution 1:1000), cleaved caspase-3 (9664, dilution 1:1000), caspase-7 (12827, dilution 1:1000), caspase-8 (9746, dilution 1:1000), caspase-9 (9508, dilution 1:1000), Bcl-2 protein family member, BID (2002, dilution 1:1000), poly(ADP-ribose) polymerase (PARP; 9542, dilution 1:1000) and cleaved PARP (5625, dilution 1:1000) were obtained from Cell Signaling Technology (Danvers, MA, USA). Antibodies against p53 (sc-126, dilution 1:1000) were purchased from Santa Cruz Biotechnology (Santa Cruz, CA, USA).

### 4.2. Plant Material

Buds of downy birch (*B. pubescens* Ehrh.) were gathered in August 2015 from trees growing in a non-protected area of the Biebrza National Park in north-eastern Poland (53° 32’ N, 22° 43’ E). A voucher specimen (No. BO-17035) was deposited with the herbarium of the Department of Pharmacognosy, Medical University of Bialystok, Poland. The birch species was identified using the approach described in [13].

### 4.3. Bud Extraction and Chemical Analysis

Downy birch buds (600 g) were ground and extracted by carbon dioxide SFE on a Waters SFE-1000F-2-FMC50 (Milford, MA, USA) system at 40 °C and a pressure of 300 bar. The dry residue from SFE was further extracted with *n*-hexane in a Soxhlet apparatus. The SFE extract (90 g) was subjected to Sephadex LH-20 gel filtration with chloroform:methanol (1:1) elution. The hexane extract (210 g) was separated on silica gel columns, eluted with hexane−chloroform, and then exposed to a chloroform−ethyl acetate step gradient (the analytical procedure is described in detail in [29]). Fractions containing triterpene compounds were subjected to further separation on polyamide columns with benzene−methanol as eluents. After multistep chromatographic separations and re-crystallization from ethanol, pure 3,4-*seco*-urs-4(23),20(30)-dien-3-oic acid (compound **1**), 3,4-*seco*-olean-4(24)-en-19-oxo-3-oic acid (compound **2**), and 3,4-*seco*-urs-4(23),20(30)-dien-19-ol-3-oic acid (compound **3**) were isolated (Figure 1). The composition of the collected fractions, as well as the purity of the isolated *seco*-acids (**1**–**3**) were determined by a gas chromatography–mass spectrometry (GC-MS) method (for details, see Supplementary Information). Mass spectra of isolated *seco*-acids, as their trimethylsilyl (TMS) derivatives, are also presented as Supplementary Information (Figures EMS-1–EMS-3). Table S1 contains the ^13^C nuclear magnetic resonance (NMR) chloroform solvent (CDCl_3_) spectra of the obtained compounds registered on a Bruker Advance II 400 spectrometer at 100 MHz. Results of NMR identification agreed with previous reports [12,16]. This was the first isolation of these triterpene *seco*-acids from *B. pubescens* buds.

### 4.4. Cell Culture and Treatment

Breast cancer MDA-MB-231 cells, colorectal cancer DLD-1 cells, gastric cancer AGS cells, glioblastoma LN229 cells, cervix cancer HeLa cells, and human skin fibroblasts CCD-25Sk were obtained from the American Type Culture Collection. Melanoma A375 cells were purchased from Sigma-Aldrich (Poznań, Poland). Cells were cultured in DMEM supplemented with 10% FBS, 100 units/mL penicillin and 100 μg/mL streptomycin in a humidified 5% CO_2_ atmosphere at 37 °C. Triterpene *seco*-acids were dissolved in dimethyl sulfoxide (DMSO) and added to the culture medium at each designated time. The final DMSO concentration in the medium for the control and compound-treated cells was 0.1%.

### 4.5. Cell Viability Assay 

Viability of cells was determined by MTT assay [30]. Cells were cultured in 96-well plates at 1 × 10^4^ cells per well for 24 h, and then treated with triterpene *seco*-acids. After 24, 48, or 72 h, MTT solution was added to each well and cells were incubated at 37 °C for 4 h. The medium was then removed and formazan crystals were dissolved in 100 μL of DMSO and 12.5 μL of Sorensen’s glycine buffer on a plate shaker. Optical density was measured in an Asys UVM340 microplate reader (Biochrom Ltd., Cambridge, UK) at 570 nm.

### 4.6. Apoptosis Assay

Cells were seeded at a density of 1 × 10^5^ cells per well in 6-well plates. After 24 h, the medium was changed and triterpene *seco*-acids were added for the next 24 h. Floating and adherent cells were collected and assayed with a Dead Cell Apoptosis Kit with annexin V-fluorescein isothiocyanate (FITC) and propidium iodide (PI) for flow cytometry (Thermo Fisher Scientific, Warszawa, Poland), according to the manufacturer’s protocol, as described in [19]. Briefly, cells were dispersed in 100 μL annexin-binding buffer containing 5 μL annexin V-FITC conjugate solution, 1 μg/mL PI, and 1 μg/mL Hoechst 33342, and incubated for 15 min at room temperature. Then, 400 μL annexin-binding buffer was added, and the cells were transferred to 96-well plates and visualized by fluorescent microscopy on a BD Pathway 855 system (Becton Dickinson, San Jose, CA, USA). Early apoptotic cells showed green and blue fluorescence, while late apoptotic cells showed green, red, and blue fluorescence.

### 4.7. Western Blot

Protein concentrations in the samples were determined by the method of Lowry et al. [31]. Proteins were resolved by sodium dodecyl sulphate-polyacrylamide gel electrophoresis (SDS-PAGE), transferred to nitrocellulose membranes, and probed with primary antibodies overnight at 4 °C. Afterwards, secondary antibodies were added for 1 h. Signals were visualized using Enhanced Chemiluminescence (ECL) reagent purchased from GE Healthcare (Waukesha, WI, USA) and recorded with a BioSpectrum Imaging System (Ultra-Violet Products, Ltd., Murray, UT, USA).

### 4.8. Statistical Analysis

The results were submitted to statistical analysis using one-way analysis of variance (ANOVA) followed by Tukey’s test, accepting *p* < 0.05 as a significant difference in comparison with controls. Half-maximal inhibitory concentration (IC_50_) values were calculated by nonlinear regression analysis using GraphPad Prism version 7.04 (GraphPad Software, San Diego, CA, USA).

## Figures and Tables

**Table 1:** IC_50_ values (μM) of compounds **2** and **3** for fibroblasts and selected cancer cell lines after 48 and 72 h of treatment.

	Compound 2 48 h	Compound 2 72 h	Compound 3 48 h	Compound 3 72 h
Fibroblasts	128 ± 5.9	84 ± 4.7	129 ± 6.8	79 ± 3.8
A.375	101 ± 4.8	76 ± 4.4	111 ± 6.0	53 ± 3.5
Ags	109 ± 5.4	69 ± 3.8	113 ± 5.1	36 ± 4.2
DLD-1	53 ± 4.9	29 ± 3.2	37 ± 4.1	26 ± 2.7
HeLa	91 ± 5.1	33 ± 4.0	118 ± 6.1	25 ± 2.0

## Supplementary Materials

The following are available online. Text S1: Gas chromatographic–mass spectrometric analysis of separated fractions and isolated compounds; Text S2: Spectroscopic and gas-chromatographic characteristics of triterpene *seco*-acids (**1**–**3**) isolated from *Betula pubescens* buds; Table S1: Data on ^13^C NMR (100 MHz, CDCl_3_) spectra of triterpene *seco*-acids (**1**–**3**).
