# Supplementary material for: Cytotoxicity of Triterpene Seco-Acids from Betula pubescens Buds
Source: Molecules. 2019 Nov 9;24(22):4060. doi: 10.3390/molecules24224060 (PMC6891332; doi:10.3390/molecules24224060)
Supplement: Supplementary file 1 [file molecules-24-04060-s001.pdf]

Supporting information

associated with article " Cytotoxicity of triterpene *seco*-acids from *Betula pubescens* buds" submitted by Ł. Szoka, V. Isidorov, J. Nazaruk, M. Stocki, L. Siergiejczyk in *Molecules*.

Corresponding author: Valery Isidorov, Forest Faculty, Białystok University of Technology, Hajnówka, Poland

This document contains the following Supporting Information:

S1 Text. **Gas chromatographic-mass spectrometric analysis of separated fractions and isolated compounds.**

S2 Text **Spectroscopic and gas-chromatographic characteristics of triterpene seco-acids (1–3) isolated from *Betula pubescens* buds**

Table S1 **Data on ^{13}C NMR (100 MHz, $CDCl_3$) spectra of triterpene *seco*-acids (1–3)**

S1 Text. Gas chromatographic-mass spectrometric analysis of separated fractions and isolated compounds

GC-MS analysis of obtained fractions and isolated compounds was performed after silanization of polar and non-volatile compounds. For this purpose, 2–3 mg of dry residue after evaporation of eluents (or 1-2 mg of isolated *seco*-acid) was put into a vial of 2 mL in volume and dissolved in 220 μL of pyridine. After adding of 80 μL of BSTFA a vial was sealed and heated for 0.5 h at 60 ^{o}C to obtain trimethylsilyl (TMS) derivatives. The GC-MS analyse of fractions was performed on a Agilent 7890A chromatograph with Agilent 5975C mass detector, on a capillary non-polar column HP-5MS (30 m x 0,25 mm x 0,25 μm) at a helium flow rate of 1 mL/min. An injection of a 1-μL sample was performed using an Agilent 7693A autosampler. The injector worked in a split (1:50) mode at an injector temperature of 300°C. The initial column temperature was 50°C, rising to 320°C, at 3 °C/min; the final temperature was held for 10 min. The ion source and quadrupole temperatures were 230°C and 150°C, respectively. Electron ionization mass spectra (EIMS) were obtained at ionization energy 70 eV.

The hexane solution of C_{10}–C_{40} *n*-alkanes were separated under the above conditions. The linear temperature programmed retention indices (I^T) were calculated from the results of separation of *n*-alkanes and silanized isolated *seco*-acids.

S2 Text. Spectroscopic and gas-chromatographic characteristics of triterpene *seco*-acids (1–3) isolated from *Betula pubescens* buds

3,4-*seco*-Urs-4(23),20(30)-dien-3-oic acid (**1**)

White crystals from EtOH. ^{13}C NMR (100 MHz, $CDCl_3$). The results of analysis are presented in Table S1. Results are in accordance with literature data [1]. EIMS-TMS: 109(100),73(92),121(67),107(65),95(60),93(52),81(50),75(48),367(46),135(41), 512(M^+); I^T = 3406.

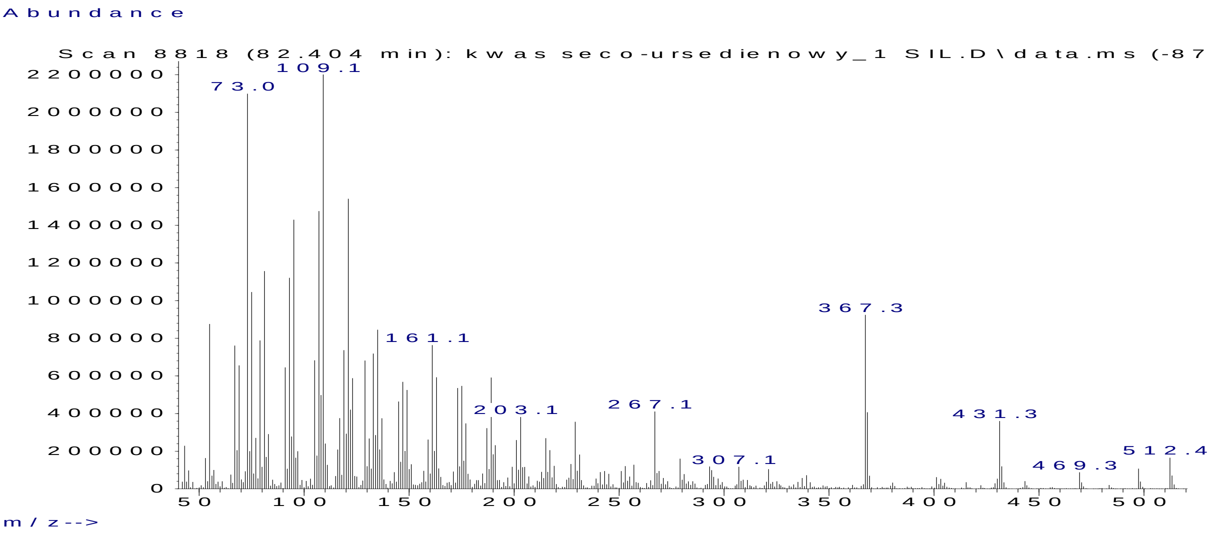

Fig. EMS-1. Mass spectrum of compound (**1**).

3,4-*seco*-Olean-4(24)-en-19-oxo-3-oic acid *(***2***)*

White powder from EtOH. The results of ^{13}C NMR analysis are presented in Table S1. and are agree with literature data [2]. TMS EIMS: 73(100),383(92),107(70), 121(66),75(63),95(63),43(62)109(61),81(51),93(50),528(M^+). I^T = 3510.

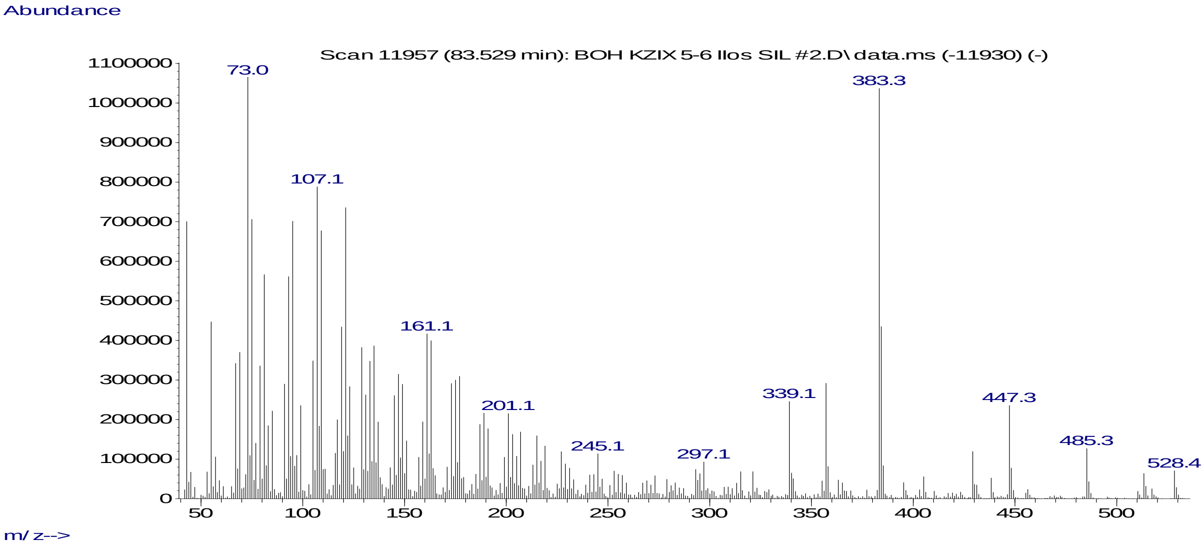

Fig. EMS-2. Mass spectrum of compound (**2**).

3,4-*seco*-Urs-4(23),20(30)-dien-19-ol-3-oic acid *(***3***)*

White crystals from EtOH. The results of ^{13}C NMR analysis are presented in Table S1. and are agree with literature data [2]. TMS EIMS: 75(100),73(64),133(37), 121(35),107(34), 119(33),109(32),105(26),95(26),187(24), 600(M$^+$). I^T = 3503.

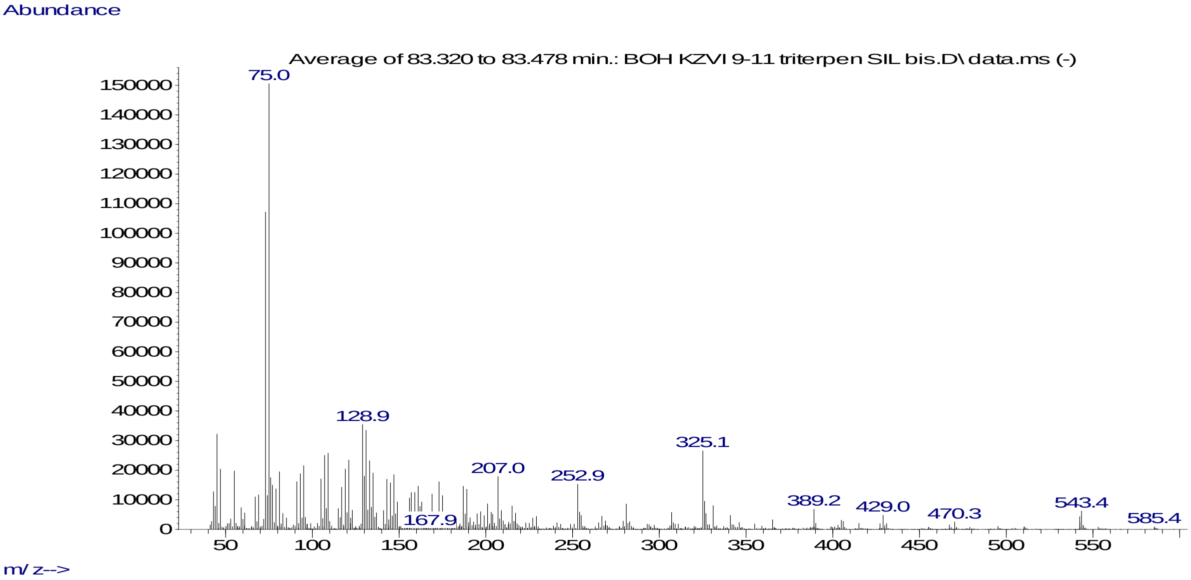

Fig. EMS-3. Mass spectrum of compound (**3**).

Table S1. The results of ^{13}C NMR (100 MHz, $CDCl_3$) of compounds **1-3** isolated from *Betula pubescens* buds.

Position	1	2	3
	δ_C		
1	34.03	33.88	34.01
2	28.23	28.07	28.12
3	176.85	178.92	179.12
4	147.54	147.47	147.52
5	50.16	50.51	50.41
6	24.45	24.61	24.57
7	32.42	32.68	32.15
8	40.42	40.68	42.52
9	39.12	40.98	41.36
10	39.02	39.24	39.27
11	21.84	21.45	21.61
12	25.95	25.36	26.54
13	40.55	34.79	33.17
14	42.29	43.62	40.77
15	26.52	27.57	37.62
16	38.06	37.72	36.52
17	34.03	43.4	36.21
18	48.47	50.35	44.48
19	39.21	213.87	73

20	154.45	54.84	151.35
21	25.42	37.84	24.63
22	38.71	40.47	37.77
23	113.03	23.23	23.19
24	22.99	113.45	113.42
25	20.04	20.03	20.24
26	15.73	15.93	15.75
27	14.44	15.22	15.65
28	19.26	20.03	16.82
29	25.15	25.44	28.06
30	106.93	20.31	107.24

1. Kashiwada, Y., Sekiya, M., Yamazaki, K., Ikeshiro, Y, Fujioka, T., Yamagishi, T., Kitagawa, S. & Takaishi, Y. Triterpenoids from the floral spikes of *Betula platyphylla* var. *japonica* and their reversing activity against multidrug-resistant cancer cells. *J. Nat. Prod.* **70,** 623–627. DOI: 10.1021/np060631s (2007).

2. Vedernikov, D.N. & Roshchin, V.I. Extractive compounds of Betulaceae family buds (*Betula pendula* Roth.): V. Composition of triterpene *seco*-acids. *Russ J Bioorg Chem* **38,** 762–768. https://doi.org/10.1134/S1068162012070229 (2012).
